# Differences between the Proliferative Effects of Human Platelet Lysate and Fetal Bovine Serum on Human Adipose-Derived Stem Cells

**DOI:** 10.3390/cells8101218

**Published:** 2019-10-08

**Authors:** Natsuko Kakudo, Naoki Morimoto, Yuanyuan Ma, Kenji Kusumoto

**Affiliations:** Department of Plastic and Reconstructive Surgery, Kansai Medical University, 2-5-1 Shin-machi, Hirakata, Osaka 573-1010, Japan; mnaoki22@kuhp.kyoto-u.ac.jp (N.M.); myy1002@163.com (Y.M.)

**Keywords:** human adipose-derived stem cells, human platelet lysate, stem cell proliferation, fetal bovine serum, signaling pathway

## Abstract

Background: Recently, human adipose-derived stem cells (hASCs) were discovered in the human subcutaneous adipose tissue. PLTMax Human Platelet Lysate (PLTMax), a supplement refined from human platelets, has been reported to have proliferative effects on bone marrow mesenchymal stem cells. The proliferative effects of PLTMax on ASCs were investigated in this study. Methods: The ASCs in DMEM (serum-free), DMEM+PLTMax (1%, 2%, 5%, and 10%), and DMEM+FBS (10%) were cultivated for two, five, and seven days. The cell growth rate was examined, BrdU incorporation, and the cell cycle and Ki-67 immunostaining were performed. The cell growth rate was investigated when each inhibitor (PD98059, SP600125, SB203580, and LY294002) was added and phosphorylation of ERK1/2, JNK, p38, and Akt were examined by western blotting. The cell surface marker of hASCs was also analyzed. Results: The cells in the PLTMax (5%) group showed significantly more proliferation compared to the cells in control (serum-free) and FBS (10%) groups, and a significant increase in the number of cells in the S phase and G2/M phase. The number of Ki-67 positive cells increased significantly in the DMEM+ PLTMax (5%) and the FBS (10%) groups. The addition of inhibitors PD98059, SP600125, SB203580, and LY294002 decreased the proliferative effects of PLTMax on ASCs. Phosphorylation of ERK1/2, JNK, p38, and Akt was observed in both the PLTMax (5%) and the FBS (10%) groups. Conclusions: For human adipose stem cells, 5% PLTMax was the optimum concentration, which showed a significantly higher proliferative effect than 10% FBS. PLTMax is a useful medium additive, which can substitute FBS. The proliferative effects of PLTMax are suggested to function via multiple signaling pathways, similar to FBS.

## 1. Introduction

Human adipose-derived stem cells (hASCs) have the potential to differentiate into adipose, bone, cartilage, tendons, nerves, and fat when cultivated under lineage-specific conditions [1,2]. The majority of ASCs exhibit fibroblastic morphological features and are easily grown under standard tissue culture conditions. Compared with bone marrow mesenchymal stem cells, hASCs are easier to obtain and carry a relatively lower donor site morbidity at harvest [3]. Due to their convenient isolation and extensive proliferative capacities in vitro, hASCs are a promising source of human stem cells for regenerative medicine.

Human platelet lysate (hPL), as prepared by repeated freeze/thaw cycles and sonication from fresh blood or outdated platelet concentrates, was found to support the proliferation of established cell lines, fibroblasts, and bone marrow mesenchymal stem cells [4,5,6]. Since our initial discovery of the enhanced proliferative effects of hPL on hASCs in 2008 [7], this phenomenon has been validated by Trojahn Kølle et al. [8] and Cervelli et al. [9]. However, the detailed mechanism of proliferation or its comparison with the effects of fetal bovine serum has not been elucidated yet. Although fetal bovine serum is the most commonly used culture supplement for hASCs at present, it has been found to increase the risk of xenogeneic infection and immune reactions as a side effect [10]. The risk of unknown infectious reagents in FBS is also a significant concern. Thus, alternatives to FBS are under investigation.

PLTMax^®^ Human Platelet Lysate (PLTMax) was a kind of hPL that has been developed as a growth factor rich supplement that is a superior alternative to fetal bovine serum (FBS) for human cell expansion. PLTMax is derived from normal human donor platelets collected at U.S. blood centers. Multiple donor units are pooled in large batch sizes and manufactured to produce a consistent product. Each donor unit was approved for human use and has been tested for infectious diseases including HIV-1, HIV-2, HCV, HBsAg, and RPR for Syphilis. To date, although there are studies showing that PLTMax can be used in cell cultures for oral mucosal epithelial cells and human corneal epithelial cell lines [11], there are no reports on its proliferative effects on hASCs and its mechanism of action.

In this study, the proliferative effects of PLTMax on hASCs were investigated by evaluating the cell proliferation, BrdU incorporation, and cell cycle changes after the addition of PLTMax to the cells. In addition, the localization of Ki-67 was assessed using immunostaining as well as activation of ERK1/2, JNK, p38, and Akt by western blotting and compared these results with that obtained from FBS treated cells.

## 2. Materials and Methods

### 2.1. Reagents and Antibodies

PLTMax^®^ Human Platelet Lysate was purchased from Merck (Darmstadt, Germany). Fetal bovine serum (FBS) was from Hyclone (Logan, UT, USA). PD98059 (an inhibitor of MEK), LY294002 (an inhibitor of phosphatidylinositol-3-kinase-AKT), and SB203580 (an inhibitor of p38) were all from Calbiochem-Novabiochem (San Diego, CA, USA). SP600125 (an inhibitor of JNK) was from Sigma (St. Louis, MO, USA). Rabbit anti-phospho-ERK1/2 was from Epitomics Inc. (Burlingame, CA, USA). Rabbit anti-phospho-AKT and rabbit anti-AKT were from Abcam (Cambridge, UK). Rabbit anti-ERK1/2 and rabbit anti-β-actin were from Cell Signaling Technology (Beverly, MA, USA). Heparin sodium injection-N was purchased from Mochida Pharmaceutical CO., LTD. (Tokyo, Japan). Antibodies of CD90, CD31, CD45, and CD34 were purchased from Beckton Dickinson Pharmingen (San Diego, CA, USA). All the other reagents, unless specified otherwise, were purchased from Sigma (St. Louis, MO, USA).

### 2.2. Isolation of hASCs

The study was approved by the Ethics Review Board of Kansai Medical University in accordance with the ethical guidelines of the Helsinki Declaration of 1975 (approval code: 2006106). All specimens were collected and used with informed consent from the volunteer donors. Adipose tissue was obtained from the patient through plastic surgery. Briefly, the adipose tissues used in this study were resected from fat mass, and not using liquid suction. The abdomen was defined as the donor site, where adipose tissues from disposed tissues from skin graft operations were extracted. hASCs were isolated using a method described previously [7,12,13]. After extensive washing with phosphate-buffered saline (PBS), the adipose tissues were cut into small pieces and then incubated with 3 volumes of 0.1% collagenase (Sigma-Aldrich, St. Louis, MO) solution with constant shaking at 40 °C for 40 min. After adding Dulbecco’s Modified Eagle Medium (DMEM) containing 10% FBS and antibiotics, it was centrifuged at 400× *g* for 3 min. After removing cellular remains through a 100 μm nylon mesh (BD Falcon, Bedford, MA, USA), the cells were incubated with DMEM containing 10% FBS and antibiotics in a dish. The primary hASCs were cultured for 4 to 5 days until they reached confluence. For all experiments, cells from passage 7 through 9 were used for the culture.

### 2.3. Cell Proliferation Assay

For the cell proliferation assays, hASCs were seeded at a density of 1.0 × 10^4^ cells/well in 24-well culture plates and incubated with the complete medium overnight. The cell medium was then replaced with serum-free DMEM. After 6 h incubation, hASCs were treated with various concentrations of PLTMax or FBS designated concentrations in serum-free DMEM for 2, 5, and 7 days. Heparin was added to the media at a final concentration of 2 U/mL for non-coagulation of medium with PLTMax. As the medium coagulated when PLTMax was added alone, the manufacturer′s protocol specified that heparin should be added to the final concentration of 2 U/mL. When inhibitors were used, they were added at 1 h before the incubation with PLTMax. Cell proliferation was determined using the Cell Counting Kit-8 (Dojindo Molecular Technologies, Kumamoto, Japan), according to the manufacturer’s instructions (*n* = 4). Absorbance was read at 450 nm on a multi-well plate reader (EnSpire 2300 Multilabel Reader; PerkinElmer, Inc., Waltham, MA, USA).

### 2.4. BrdU Incorporation Assay

The cells were seeded at a density of 2 × 10^3^ cells/well in 96-well culture plates containing a complete medium. After overnight incubation, the hASCs were first starved in a serum-free DMEM for 6 h. These cells were then treated with PLTMax in the serum-free DMEM for 48 h. Inhibitors were added at 1 h before the incubation withPLTMax. Quantification of cell proliferation was determined using the Cell Proliferation ELISA BrdU kit (Roche), according to the manufacturer’s instructions (*n* = 4).

### 2.5. Cell Cycle Assay

The MuseTM Cell Cycle reagent included propidium iodide (PI) as the binding reagent (intercalator) for DNA. Fluorescence intensity of an intercalated fluorescent substance represents the DNA amount and the cell cycle stage. Muse Cell Cycle Reagent was included in the Muse Cell Cycle Kit. hASCs (1 × 10^6^) were seeded in a 10-cm culture dish containing complete medium and cultured overnight. The medium was then replaced with serum-free DMEM. After starvation for 6 h, the cells were then treated with the reagents with designated concentrations for 48 h. Treated cells were collected by trypsinization. After washing with ice-cold PBS twice, cells were fixed in 70% ethanol at −20 °C for 3 h. Based on the manufacturer’s instructions, the fixed cells were then stained with MuseTM Cell Cycle reagent (200 μL) in the dark at room temperature for 30 min. Cell cycles were analyzed by flow cytometric quantification of their DNA by MuseTM Cell Analyzer (Millipore, Hayward, CA, USA) (*n* = 6 in each group).

### 2.6. Cell Surface Marker of hASCs

The phenotypical characterization of the ASCs was analyzed using BD FACSCalibur (Becton-Dickinson, Heidelberg, Germany) and accompanying software. At the 7th generation, the cells were detached with trypsin-EDTA, washed with phosphate-buffered saline (PBS), and immediately stained with the following labeled antibodies: CD90, CD31, CD45, CD34. Regarding ASCs after 48 h of PLTMax culturing, 1 × 10^6^ cells were prepared per measurement, and the positive cell rate was analyzed (*n* = 3).

### 2.7. Immunofluorescence Confocal Microscopy

The cells were plated in Celldesk LF (Sumitomo Bakelite Co Ltd., Tokyo, Japan) with serum-free DMEM for 24 h before stimulation. Subsequently, cells were stimulated with PLTMax or FBS for 48 h. The cells were fixed with 4% formaldehyde solution for 15 min and then washed thrice with PBS. Following that, 0.2% Triton X-100 was added to the cells, incubated for 5 min, and then washed thrice with PBS. Further, the cells were blocked using Protein Block Serum-Free solution (Dako Japan Inc., Tokyo, Japan) for 1 h, stained using Phalloidin, and conjugated using Rhodamine X (FUJIFILM Wako Pure Chemical Corporation, Osaka, Japan) for 30 min. Phalloidin is a bicyclic peptide belonging to a family of toxins isolated from the deadly Amanita phalloides mushroom, and it is commonly used in imaging applications to selectively label F-actin. In this study, rhodamine-labeled phalloidin was used. After washing thrice with PBS, the cells were incubated with the Ki-67 rabbit monoclonal antibody (Cell Signaling Technology) at a concentration in 1:400 at 4 °C overnight. Then, anti-rabbit IgG (H+L) and F(ab′)2 fragment (Alexa Fluor^®^ 488 Conjugate; Cell Signaling Technology) was added and incubated for 2 h. After washing thrice with PBS, ProLong^®^ Gold Antifade Reagent with DAPI (Cell Signaling Technology) was added to the cells and enclosed. Cells were viewed using a laser scanning confocal microscope (LSM510-META, Carl Zeiss, Jena, Germany).

### 2.8. Western Blot Analysis

The cells were treated with indicated compounds and lysed. Extracted cellular proteins (20 µg) were separated by sodium dodecyl sulfate-polyacrylamide gel electrophoresis (SDS-PAGE) and then transferred to a polyvinylidene difluoride (PVDF) membrane. In the SDS-PAGE step of the Western blotting, the cellular protein was isolated under reducing conditions using β-mercaptoethanol. The membrane was first blocked with Blocking One-P reagent (Nacalai Tesque, Kyoto, Japan) for 30 min at room temperature, and then incubated with primary antibodies at 4 °C overnight. After washing with PBS (-), the membranes were incubated with a peroxidase-linked secondary antibody at room temperature for 30 min. The labeled proteins were detected with the enhanced chemiluminescence using the Prime Western blotting detection system (GE Healthcare).

### 2.9. Statistical Analysis

The Mann-Whitney *U* test was used for comparisons between groups, with *p* < 0.05 being regarded as significant. Data are presented as means ± S.D.

## 3. Results

### 3.1. PLTMax Stimulated Proliferation of hASCs

**Figure 1:**
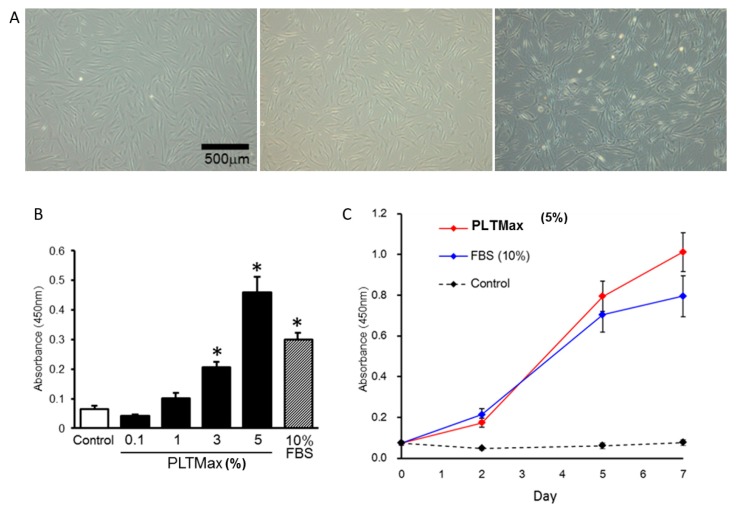
(**A**) The morphology of human adipose-derived stem cells (hASCs) observed by phase-contrast microscopy. Representative images of the cultures in the control group (no serum), fetal bovine serum (FBS) (10%), PLTMax (5%) after two days are shown. (**B**) The proliferation of hASCs cultured using 1%, 3%, and 5% PLTMax. The 3% PLTMax group showed significantly higher proliferation compared to the control group (no serum) and the 5% PLTMax group showed higher proliferation compared to the 10% FBS group. * *p* < 0.05 (**C**) The chronological changes in the proliferation of cells cultured on days 2, 5, and 7 in the control group, FBS-treated (10%), and PLTMax-treated (5%) cells. On days 5 and day 7, the cells treated with PLTMax (5%) showed higher proliferation compared to FBS (10%).

The cell growth stimulated by PLTMax was confirmed by observation with phase-contrast microscopy. Compared to the control (no serum) and FBS (10%) groups, the cells treated with PLTMax (5%) were slightly elongated, and the nucleus was visible (Figure 1A).

Cell proliferation was increased by treatment with the 1%, 3%, 5% PLAMax group, and the FBS (10%) group (*p* < 0.01 vs. control). Among them, the cells in the 5% PLTMax group showed the highest proliferation, even higher than the 10% FBS group (Figure 1B). When the cells were incubated in PLTMax (5%), FBS (10%), and control (no serum) conditions for 2 to 7 days, high proliferation in the PLTMax (5%) group and FBS (10%) group on the 5th and 7th days was observed, while no proliferation was observed in the control group, even on the 7th day (Figure 1C).

### 3.2. PLTMax Promoted Cell Cycle Transition from G0/G1 to S Phase

**Figure 2:**
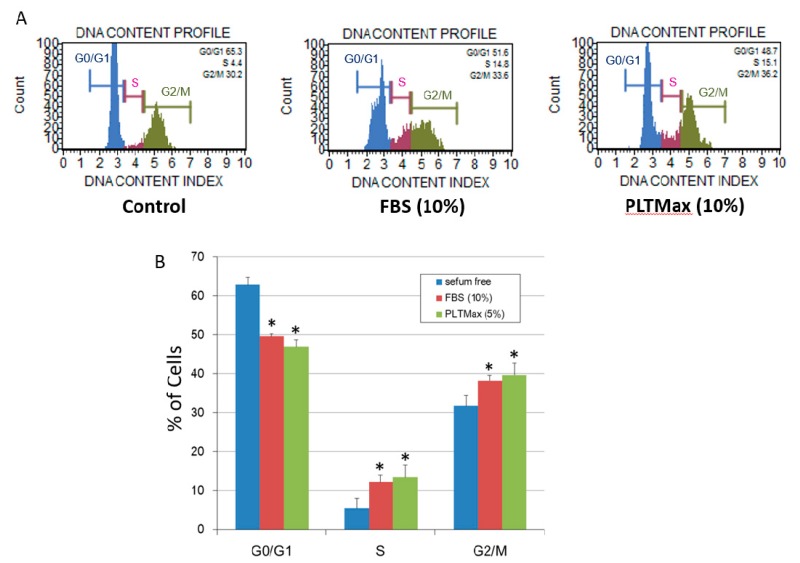
(**A**) Histogram of the typical cell cycle of hASCs from the control group (no serum), FBS (10%) group, and PLTMax (5%) group, as evaluated using flow cytometry. (**B**) The percentage of hASCs cultured in the PLTMax (5%) group in the G0/G1 phase, S phase, and G2/M phase. Compared to the control group (no serum), the cells in the FBS (10%) and PLTMax (5%) group showed a significantly increased percentage of cells in the S phase and G1/G2 phase. (*n* = 6) * *p* < 0.05.

In the FBS (10%) group and the PLTMax (5%) group, a decrease in the percentage of cells in the G0/G1 period compared to that in the control group was observed. A histogram of the typical cell cycle, as evaluated by flow cytometry is shown in Figure 2A. The cells in the G0/G1 period were 62.87% ± 1.93% in the control group, 49.62% ± 2.60% in the FBS (10%) group, and 46.89% ± 2.72% in the PLTMax (5%) group, respectively. The cells in the S period were 5.41% ± 0.61% in the control group, 12.16% ± 1.77% in the FBS (10%) group, and 13.41% ± 1.44% in the PLTMax (5%) group, respectively. Further, the cells in the G2/M period were 31.71% ± 1.81% in the control group, 38.19% ± 3.11% in the FBS (10%) group, and 39.69% ± 3.05% in the PLTMax (10%) group, respectively. In the G0/G1 period, the FBS (10%) group and the PLTMax (5%) group showed significantly lower cell counts than the control group. In the S period and the G2/M period, the FBS (10%) group and the PLTMax (5%) group showed significantly higher cell counts than the control group, and among them, the cell count in the PLTMax (5%) group was the highest (Figure 2B).

### 3.3. Effect of PLTMax on the Number of Ki-67 Positive Cells

**Figure 3:**
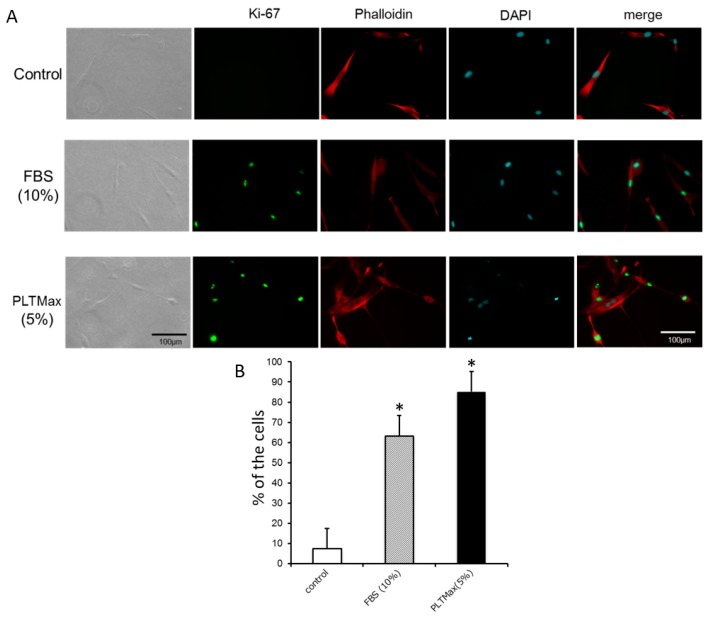
(**A**) Typical images of hASCs cultured without serum (control), with FBS (10%), and PLTMax (5%) immunostained for Ki-67, and with phalloidin and DAPI, or triple stained. Ki-67 staining was localized in the nucleus. (**B**) Percentage of Ki-67 positive cells. Compared to the control group, FBS (10%) and PLTMax (10%) showed a significantly increased percentage of Ki-67 positive cells. PLTMax (5%) showed the highest percentage among the 3 groups. * *p* < 0.05.

Fluorescent triple immunostaining was performed, which included Ki-67 fluorescent staining, Phalloidin staining, and DAPI nuclear staining, for the cells in the control group, the FBS (10%) group, and the PLTMax (5%) group. Ki-67 staining was found to be localized in the nucleus (Figure 3A). The percentage of Ki-67 positive cells was 7.46% ± 11.49% in the control (no serum) group, 63.33% ± 12.92% in the FBS (10%) group, and 85.19% ± 7.01% in the PLTMax (5%) group (Figure 3B), respectively. In the FBS (10%) group and the PLTMax (5%) group, the number of Ki-67 positive cells was significantly higher than that in the control group with the PLTMax (5%) group showing the highest number.

### 3.4. Effect of PLTMax on the Cell Surface Marker of hASCs

The effects of PLTMax on the cell surface marker of hASCs after 48 h of PLTMax culturing were analyzed. ASCs were CD90 (98.60% ± 0.99%), CD31 (0.20% ± 0.28%), CD45 (7.10% ± 3.77%), CD34 (5.77% ± 0.70%) at the seventh generation. After PLTMax culturing, they were CD90 (99.33% ± 1.15%), CD31 (0.33% ± 0.31%), CD45 (7.27% ± 7.64%), and CD34 (9.20% ± 4.69%). There was no significant difference between the cell surface markers before and after culturing.

### 3.5. PLTMax Activated ERK1/2, AKT, and JNK Signaling Pathways

**Figure 4:**
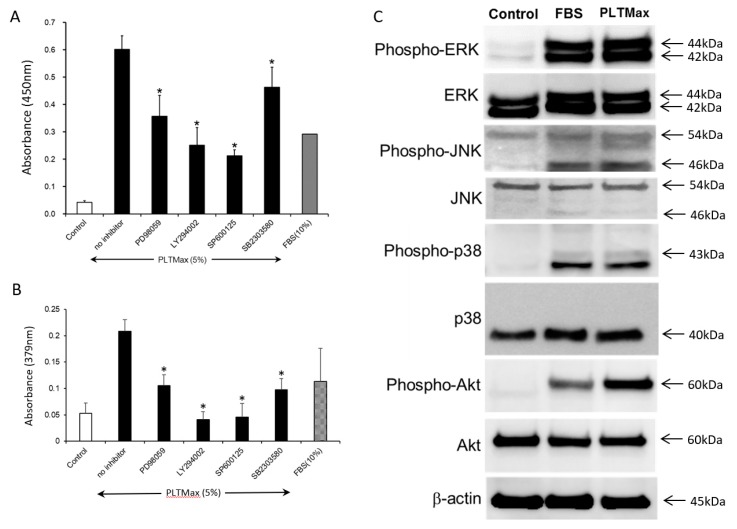
(**A**)The effect of various inhibitors (PD98059, LY294002, SP600125, and SB2303580) added in the presence of PLTMax (5%) on the proliferation of hASCs. The proliferative effect of PLTMax was partially inhibited by all the inhibitors. * *p* < 0.05 (**B**) The effect of various inhibitors (PD98059, LY294002, SP600125, and SB2303580) added in the presence of PLTMax (5%) on the BrdU incorporation of hASCs. The proliferative effect of PLTMax was partially inhibited by all the inhibitors. * *p* < 0.05 (**C**) MAP kinase-related proteins and phosphorylation seen in the hASCs in the control (no serum), FBS (10%) and PLTMax (5%) groups. The protein expressions of phospho-ERK, phospho-JNK, phospho-p38, and phospho-Akt were observed in FBS (10%) group and PLTMax (5%) group.

The cells were treated with ERK1/2 inhibitor (PD98059, 50 µM), PI3K/AKT inhibitor (LY294002, 10 µM), JNK inhibitor (SP600125, 20 µM) and p38 inhibitor (SB203580, 20 µM), to examine the signaling pathways involved in the stimulation of hASCs by PLTMax. The PLTMax induced proliferation of cells was suppressed by PD98059, LY294002, SP600125, and SB203580 (Figure 4A). The PLTMax induced BrdU incorporation of cells was also suppressed by PD98059, LY294002, SP600125, and SB203580 (Figure 4B). The signaling pathways were further analyzed in the hASCs by Western blot. Phosphorylation of ERK1/2, JNK, p38, and AKT pathways was increased with the stimulation of either PLTMax or FBS. In the control group, phosphorylation of ERK1/2, JNK, p38, and AKT pathways did not occur. Thus, the stimulation of cell growth by PRP was mediated through multiple signal pathways (Figure 4C).

## 4. Discussion

PLTMax is a medium supplement that can be used as an FBS replacement. In this study, it was shown that 5% PLTMax is the optimal concentration and its proliferative effect was significantly higher compared to 10% FBS. PLTMax may have exerted its proliferative effect via multiple signaling pathways, similar to FBS.

Mesenchymal stromal stem cells (MSCs) can potentially differentiate into mesenchymal cells, including osteoblasts, adipocytes, muscle cells, and chondrocytes, and they have been considered for their application in regenerative medicine. Although the studies conducted with MSCs thus far have focused on the cells established from the bone marrow, recent studies have revealed that these cells can be established from several other tissues, such as cord blood, placenta, and adipose tissues [14]. Among them, adipose tissue contains a higher number of MSCs compared to the bone marrow, and the MSCs established from the adipose tissues proliferate rapidly [15]. Although the studies conducted with MSCs thus far have focused on the cells established from the bone marrow, recent studies have revealed that these cells can be established from several other tissues, such as cord blood, placenta, and adipose tissues. Among them, adipose tissues contain a higher number of MSCs compared to the bone marrow, and MSCs established from the adipose proliferate rapidly. Therefore, adipose-derived MSCs are garnering a lot of interest [16]. In order to clinically use hASCs in regenerative medicine, hASCs have to be prepared from a small number of adipose tissues and cultured ex vivo on a large scale to minimize invasiveness in the donor. To date, FBS has been generally used as a supplement to culture hASCs. However, concerns related to the use of FBS, such as bovine spongiform encephalopathy infection and certain other unidentified infections, risks of xeno-immunization against bovine antigens, the transmission of pathogens, and ethical issues associated with crude methods of FBS collection [17,18,19,20,21,22] have led to the exploration of supplements that can substitute FBS.

Platelets play an essential role not only in primary hemostasis but also in wound healing and tissue regeneration. The α-granules of the platelets contain several chemokines and growth factors, such as isoforms of platelet-derived growth factor (PDGF), transforming growth factor-β (TGF-β), insulin-like growth factor (IGF), vascular endothelial growth factor (VEGF), epidermal growth factor (EGF), and basic fibroblast growth factor (bFGF) [10]. To our knowledge, our study was the first to report that platelet-rich plasma prepared from whole blood contains high levels of PDGF and TGF-β and has a proliferative effect when added to hASCs. Human platelet lysate (hPL) is created from single or pooled donor-derived platelets isolated from the whole blood or by apheresis, and it is distributed in standard platelet collection bags. Several researchers have reported the proliferative effect of hPL on bone marrow MSCs. The proliferative effect of hPL (5% to 10%) on bone marrow MSCs was superior to that of FBS [6,23,24]. However, there are currently no studies investigating the mechanism underlying the effect of hPL on hASC proliferation.

PLTMax is a commercialized human platelet lysate product manufactured by Merck, created from the whole blood of American donors, which has been checked for infections. Studies on cell proliferation using PLTMax were recently conducted using human corneal epithelial cells [11,25] and oral mucosal epithelial cells [26]. Huang et al. reported that in human corneal epithelial cells, FBS seemed to have a higher proliferative effect compared to PLTMax. In addition, they reported that a higher concentration (10%) of PLTMax showed stronger inhibitory effects on cell proliferation compared to FBS [11]. In this study, the hASCs treated with 5% PLTMax showed significantly higher proliferation compared to those treated with 10% FBS. Different cell-types can show different reactions to PLTMax. PLTMax might be a better supplement for the mass cell culture of hASCs compared to FBS. Hsueh et al. reported that they succeeded in creating an oral mucosal epithelial cell sheet without animal-derived components with the addition of PLTMax instead of FBS [26]. Therefore, it is possible to create a cell sheet with hASCs using PLTMax.

In this study, it was found that PLTMax increased the percentage of cells in the S phase and G2/M phase of the cell cycle, as well as the Ki-67 positive cells. While Ki-67 is present in all cell cycle (G1, S, G2, and M) phases in proliferating cells, the G0 phase does not occur when cell proliferation is intermitotic. The cellular content of Ki-67 markedly increases during cell cycle progression throughout the synthetic phase (S phase) of the cell cycle [26]. Therefore, the high nuclear expression of Ki-67 in ASCs that were treated with PLTMax indicates an enhanced proliferative effect of the additive. This proliferative effect is suppressed by the addition of ERK, JNK, p38, and Akt inhibitors, suggesting the involvement of multiple signaling pathways. Studies conducted by both Chen et al. and Huang et al. revealed that PLTMax contains PDGF, TGF-β, and EGF. These factors might be involved in inducing the proliferative effect via several signal pathways in the treated hASCs [11,25]. Hence, in the future, it is necessary to investigate the proliferative factors and cytokines affected by the addition of PLTMax and to identify the factor that is most closely associated with cell proliferation.

Lensch M. et al. [27] published an article about the effect of commercially available synthetic media designed for adipose-derived stem cell expansion, including PLTMax Human Platelet Lysate (acquired from Sigma-Aldrich). Lensch M. et al. also performed immunophenotypic characterization of ASCs and evaluated their ability to differentiate into osteoblasts and adipocytes after treatment with PLTMax. They reported that PLTMax had a proliferative effect on the ASCs. In addition, they reported that cell culture using PLTMax up-regulated CD105, which is a cell surface marker, by 21%, while it has no effect on CD73 and CD90. However, the PLTMax that was used in the study by Lensch M. et al. [27] was manufactured by Sigma-Aldrich, while the PLTMax used in our study was manufactured by Merck. Thus, it is uncertain if their composition is similar due to the difference in manufacturers. Furthermore, they did not clearly mention the percentage concentration of PLTMax added to the medium in their report. On the other hand, other researchers have also reported the proliferative effect of PLTMax on ASCs. In a study by Morten et al., they added a 5% concentration of PLTMax to the ASCs and compared it with the FBS culture, similar to our study. They reported that while the levels of CD105 and CD90 hardly changed at P0, the expression of CD19 was higher (~30%) in the ASCs cultured in FBS medium at P0 and not in the PLAMax medium. They also reported that the expression of the secondary ASCs marker, CD 36 generally varied between 40% and 60% for PLTMax-supplemented ASC cultures, which decreased slightly after passaging. Interestingly, they investigated the genetic stability of ASCs till the 5th passage and they did not observe any imbalanced chromosomal rearrangements. These results are consistent with our study results. Moreover, recently it has been reported that the expression of ASC markers might be affected by the in vitro culture conditions and the passage number [16]. Therefore, the genetic stability and aging after culture with PLTMax should be investigated in the future for better understanding.

Previous studies have made efforts to develop serum-free products that can provide all the essential nutrients and the growth factors to maintain physiological function and to promote cell proliferation [28,29,30,31]. However, most of these serum replacements could not support cell growth [32]. Therefore, as an alternative to animal serum for cell proliferation, autoserum and allogeneic human serum have been investigated. On the one hand, few studies have reported that human serum improved cell growth, and on the other hand, some studies showed that it suppressed cell growth [33]. Our study showed that human serum promoted the proliferation of adipose-derived stem cells. However, certain concerns regarding the clinical applications, such as difficulty in collection and processing and the difference in the quality of autoserum from patient to patient, might hamper the standardization of culture conditions. In addition, the optimum amount of autoserum required for the expansion of cultures exceeds the amount that a single donor can provide [29]. In the future, we plan to investigate methods for collection and processing that can overcome these issues.

PLTMax has been suggested to be a more effective cell culture supplement for the culture of hASCs compared to FBS. If the culture of hASCs using hPL prepared from autologous platelets is successful, it would be possible to conduct an efficient mass cell culture without the risk of infection by unknown pathogens derived from animals. hPL is a supplement that might replace FBS in the future. Further studies need to be conducted to obtain the basic data for its application in regenerative medicine, particularly data regarding the changes in the cell surface markers, differentiation potency, and presence/absence of karyotype abnormality.
